# SELF-TAILORED SUCCESSFUL AGING: OLDER ADULTS’ POINTS OF VIEW

**DOI:** 10.1093/geroni/igac059.3018

**Published:** 2022-12-20

**Authors:** Michal Tsadok Cohen, Sara Rosenblum, Sonya Meyer

**Affiliations:** Haifa University, Haifa, Hefa, Israel; Haifa University, Haifa, Hefa, Israel; Ariel University, Ariel, HaMerkaz, Israel

## Abstract

The study aimed to map out the main constructs involved in aging successfully, as perceived by older adults, and highlight the importance of self-management. This qualitative study was comprised of eighteen interviewees and sixty participants across nine focus groups, including: Independent older adults aged 65 to 84 years living at home, health professionals working with older adults, and family members of older adults. Participants shared experiences concerning their everyday occupations and routines. Focus groups were recorded, transcribed verbatim, and qualitatively analyzed, using the constructivist grounded theory principles.

Results: Three successful aging theoretical categories emerged: self-management abilities, daily lifestyle, and individual differences. An example for self-management abilities can be described by a 67-year-old man’s son, who said: “He is a very busy man, he doesn’t have any commitments, but he always creates things for himself to do”. Results revealed diversity in how older adults perceive successful aging. For example, a 68-year-old woman said: “Retirement is like heaven, I enjoy every moment, there’s a lot to do”, whereas a 69-year-old man claimed: “Nobody needs you anymore… I retired a year ago and I still haven’t succeeded in organizing a daily schedule”. Additional results and quotes reflecting the categories will be represented.

Conclusion: Older adults who succeed in managing their daily life, and experience control of their own life, delineate successful aging. The diversity accentuates that each older adult needs to tailor their own self fitted “suit” to wear as they stride successfully into their aging years.

